# The potential causal relationship between fat mass in different body areas and low back pain: Findings from NHANES and Mendelian randomization studies

**DOI:** 10.1097/MD.0000000000043891

**Published:** 2025-08-15

**Authors:** Jingyan Yang, Yupei Cheng, Renjun Huang, Yuyang Zhao, Chaoyi Wang, She Ma, Jingjie Huang, Yuyan Chen, Chaoran Wang, Qian Yu, Bangqi Wu, Dong Yu

**Affiliations:** a Beijing University of Chinese Medicine Third Affiliated Hospital, Xiaoguanjie, Beling, Chaoyangqu, China; b First Teaching Hospital of Tianjin University of Traditional Chinese Medicine, Tianjin, China; c National Clinical Research Center for Chinese Medicine Acupuncture and Moxibustion, Tianjin, China.

**Keywords:** fat mass, low back pain, Mendelian randomization, NHANES

## Abstract

The purpose of this study is to investigate and discuss the potential relationship between fat distribution in different body regions and low back pain (LBP). We used 2 research methods to study the impact of fat distribution on LBP. First, we conducted a cross-sectional study using National Health and Nutrition Examination Survey data to examine the relationship between fat levels in the trunk and lower limbs and LBP. Second, we employed a two-sample Mendelian randomization approach to investigate the causal relationship between trunk fat, lower limb fat, and LBP. In the cross-sectional study, the results of multivariable logistic regression analysis showed that for every unit increase in trunk fat mass (FM) (kg), the risk of LBP increased by 2.8% (odds ratios, OR = 1.028, 95% confidence intervals [CI] [1.021, 1.034], *P* < .00001). Similarly, there was a positive correlation between right leg FM, left leg FM, and LBP (fully adjusted model: OR = 1.075, 95% CI [1.053, 1.097], *P* < .00001; OR = 1.076, 95% CI [1.053, 1.099], *P* < .00001). Mendelian randomization analysis indicated that an increase in trunk FM had a detrimental effect on LBP (OR = 1.296, 95% CI [1.207–1.392], *P* = 9.86 × 10^-13^), while an increase in left lower limb FM had a protective effect on LBP (OR = 0.672, 95% CI [0.604–0.748], *P* = 2.61 × 10^-13^), and an increase in right lower limb FM also had a protective effect on LBP (OR = 0.655, 95% CI [0.590–0.728], *P* = 3.32 × 10^-15^). The study revealed a positive causal relationship between trunk FM and LBP. However, the causal relationship between FM in the lower extremities and LBP remains controversial. Nonetheless, investigating lower extremity FM as a potential protective factor in depth is a direction worth focusing on in the future.

## 1. Introduction

In recent years, the incidence of low back pain (LBP) has been increasing annually, showing a trend towards generalization and younger age groups. This trend has caused significant disruption to people’s work and daily lives.^[1,2]^ Mainstream research suggests that the primary cause of LBP is related to degenerative changes in the spine. The main pathological basis is due to the aging and degeneration of intervertebral discs, leading to a gradual loss of their original function.^[3]^ Further research indicates that obesity is one of the key pathogenic factors leading to intervertebral disc degeneration.^[4]^ Existing mechanistic studies have found that fat infiltration in corresponding regions of the trunk, along with excessive stress, as well as adipocyte-derived factors like leptin and adiponectin, are key factors inducing inflammation in the lumbar region and thus causing LBP.^[5,6]^ Abnormal expression of fat content in intervertebral disc tissues leads to an initial inflammatory environment and metabolic disruption, which is one of the important pathogenic pathways of LBP.^[7]^

Fat mass (FM) is widely used to assess the risk of various diseases, but its use in evaluating LBP is less reported, given its strong correlation with body mass index (BMI). Recent studies have confirmed that local fat thickness can serve as a reliable analytical tool for assessing the relationship between LBP and degenerative changes in the spine, and it can be a more intuitive marker of obesity levels.^[8]^ Wang M et al found through lumbar spine magnetic resonance imaging that compared to BMI, measures such as abdominal circumference and sagittal abdominal diameter were more reliable tools for assessing the degree of intervertebral disc degeneration in LBP patients after Pfirrmann grading.^[9]^ The above study illustrates that besides FM itself, the different distribution of fat can also have varying effects on the body, but the clear causal relationship still requires further exploration.

When studying causative factors and disease risks, randomized controlled trials hold high reference value. However, they are limited in their ability to be universally conducted due to various clinical factors. Therefore, in this study, to further explore whether there is a certain connection between FM in different body areas and LBP, we first conducted a cross-sectional study using relevant data from the National Health and Nutrition Examination Survey (NHANES) database to investigate the potential link between FM in different body areas and LBP. The database includes a wealth of interviews, physical examinations, laboratory tests, and other information regarding both adults and children in the United States, providing strong population representativeness. Additionally, we further evaluated the causal relationship between FM and the risk of LBP using Mendelian randomization (MR) methods. This method utilizes genetic variations as instrumental variables (IVs),^[10]^ following Mendel law, which states that alleles are randomly distributed and fixed during embryonic development. This principle helps to eliminate confounding factors’ impact on results, thereby strengthening the strength of evidence for causal inference in research,^[11,12]^ achieving a purpose similar to simulating a RCT. The study combines clinical and Mendelian research characteristics, excluding total body FM and upper limb FM, and selects trunk and bilateral lower limb FM along with LBP as exposure factors and outcome indicators for NHANES and MR analysis, aiming to clarify the potential causal relationship between different FM distributions and LBP.

## 2. Materials and methods

### 2.1. Study design

We used 2 research methods to study the impact of different FM distributions on LBP. Firstly, we conducted a cross-sectional study using data collected from NHANES between 1999 and 2004 to investigate the relationship between trunk and lower limb FM and LBP. The NHANES database samples representative non-institutionalized populations in the United States every 2 years as 1 cycle to describe health status and disease burden. The data from these cross-sectional surveys are sourced from the National Center for Health Statistics and can be accessed at https://www.cdc.gov/nchs/index.htm. We utilized data from 3 consecutive two-year cycles spanning from 1999 to 2004. Next, we employed a two-sample MR method to examine the causal relationship between trunk FM, bilateral lower limb FM, and LBP. The study design and the 3 main hypotheses of MR are illustrated in Figure S1, Supplemental Digital Content, https://links.lww.com/MD/P672. The study strictly adheres to the guidelines provided in the Strengthening the Reporting of Observational Studies in Epidemiology using Mendelian Randomization (Supplementary STROBE-MR Checklist) and Strengthening the Reporting of Observational Studies in Epidemiology (Supplementary STROBE-cross-sectional studies Checklist).

### 2.2. Data sources

#### 2.2.1. Data for NHANES study

The current cross-sectional study utilizes NHANES data from 1999 to 2004 obtained from the National Center for Health Statistics. The study includes individuals aged 20 or above who have completed interviews, excluding those lacking data on FM, LBP, or covariates. The main outcome, LBP, is considered a binary variable defined as whether LBP occurred in the past 3 months. The exposure variable, FM, is a continuous variable measured in kilograms (kg), including trunk FM, right leg FM, and left leg FM. The study aims to reduce bias by assessing potential covariates based on existing literature.^[13–15]^ These covariates include age, gender, race, household income, education level, smoking status, alcohol status, BMI, physical activity level, as well as hypertension and diabetes. The race classifications in the study include Non-Hispanic White, Non-Hispanic Black, Other Hispanic, Mexican American, and Other races. Based on the poverty income ratio (PIR),^[16]^ family income is divided into 3 groups: low (≤1.3), middle (1.3–3.5), and high (> 3.5). Education level is categorized as follows: below high school, high school diploma (including GED), and above high school. The smoking status is divided into 3 groups: never: smoked <100 cigarettes in life, former: smoked more than 100 cigarettes in life and smoke not at all now, now: smoked moth than 100 cigarettes in life and smoke some days or every day. The drinking status is divided into 5 groups: never (had < 12 drinks in lifetime); former (had ≥ 12 drinks in 1 year and did not drink last year, or did not drink last year but drank ≥ 12 drinks in lifetime); mild (≥1 drinks per day for females, ≥2 drinks per day for males); moderate (≥2 drinks per day for females, ≥3 drinks per day for males, or binge drinking ≥ 2 days per month); heavy (≥3 drinks per day for females, ≥4 drinks per day for males, or binge drinking [≥4 drinks on same occasion for females, ≥5 drinks on same occasion for males] on 5 or more days per month).^[17]^ BMI (kg/m^2^) is a standardized measure calculated based on weight and height measurements. Physical activity level is divided into 4 groups: not walking regularly; frequently walks but doesn’t need to carry or lift things often; light load, frequently climbs stairs or hills; engaged in heavy work or lifting heavy objects. Diagnose the presence or absence of hypertension through the calculation of mean blood pressure. Average blood pressure was calculated by the following protocol: the diastolic reading with zero is not used to calculate the diastolic average. If all diastolic readings were zero, then the average would be zero. If only 1 blood pressure reading was obtained, that reading is the average. If there is more than 1 blood pressure reading, the first reading is always excluded from the average. Diabetes is divided into 3 categories: diabetes mellitus (DM), impaired fasting glucose (IFG), No. The diagnostic criteria for diabetes are: doctor told you have diabetes, glycohemoglobin HbA1c (%) ≥6.5, fasting glucose (mmol/l) ≥ 7.0, random blood glucose (mmol/l) ≥ 11.1, 2-hour OGTT blood glucose (mmol/l) ≥ 11.1, use of diabetes medication or insulin.

#### 2.2.2. Data for MR

In the MR study, we used LBP as the outcome variable and selected single nucleotide polymorphisms (SNPs) significantly correlated with trunk and bilateral lower limb FM as IVs. Genetic data for trunk and bilateral lower limb FM were obtained from the IEU GWAS database. The genetic data for left lower limb FM and right lower limb FM were sourced from the Neale lab’s GWAS study in 2017. The dataset for left lower limb FM comprises 331,275 samples and 10,894,596 SNPs, while the dataset for right lower limb FM comprises 331,293 samples and 10,894,596 SNPs. The data for trunk FM is sourced from the MRC Epidemiology Unit’s 2018 GWAS meta-analysis, with a sample size of 454,588 and 9861,867 SNPs. Genetic variation data for LBP is sourced from the FinnGen consortium (https://www.finngen.fi), which collects and analyzes genomic and health data from 500,000 Finnish participants. The LBP accession number is: finngen_R9_M13_LOWBACKPAI, which includes 29,329 cases and 270,964 controls of European descent. These cases are diagnosed based on the World Health Organization’s International Classification of Diseases Inclusion Criteria (ICD-10 M54.5, ICD-9 724.2, ICD-8 728.70).

### 2.3. Selection of IVs

The study adopts the following 3 key assumptions to ensure the reliability of MR analysis: ① the relevance assumption: the instrumental variable is strongly correlated with the exposure factor; ② the independence assumption: the IVs is independent of any confounding factors related to the exposure factor and outcome variable; ③ the exclusion restriction assumption: the IVs can only affect the outcome through the exposure factor.^[18,19]^ To meet the relevance assumption, which states that the IVs selected is strongly correlated with the exposure factor, this study employed a threshold of *P* < 5 × 10^-8^ to screen for genome-wide significantly different SNPs. Additionally, the clumped function was used to remove SNPs in linkage disequilibrium (*r*^2^ < 0.001, 10,000kb). To meet the exclusion restriction assumption and reduce bias from weak IVs, this study filtered out weak IVs with an F-value < 10 using the formula F = [*R*^2^ × (N - 1 - K)]/[K × (1 - *R*^2^)],^[20,21]^ where N represents the sample size in the exposure factor GWAS study, K is the number of SNPs selected as IVs after screening, and *R*^2^ denotes the proportion of variance explained by SNPs in the exposure database (*R*^2^ = 2 × EAF × (1 - EAF)×β^2^, where EAF is the allele frequency of the mutation,^[22]^ and β is the effect value of the allele^[23,24]^). The MR-Egger regression was then employed to test the pleiotropy of SNPs. If *P* < .05, it indicates the presence of pleiotropy, and the SNP does not meet the requirements of an IV.^[25,26]^ The MR pleiotropy residual sum and outlier (PRESSO) test were then conducted to assess and adjust for horizontal pleiotropy outliers. The outlier-corrected method within the model was used to remove outliers from the IVs. This rigorous screening process resulted in the selection of IVs for the MR analysis.

### 2.4. Statistical analysis

In the NHANES study, we first described the baseline characteristics of the overall sample, the LBP group, and the non-LBP group. Continuous variables were presented as means and standard deviations, while categorical variables were presented as frequencies and percentages. Differences between the LBP and non-LBP groups were assessed using *t* tests for continuous variables and chi-square tests for categorical variables. Using multivariable logistic regression analysis, we examined the relationship between FM (both continuous and quartiles) and LBP. Odds ratios (ORs) and 95% confidence intervals (CIs) were calculated to assess the strength of the association. The crude model was adjusted for no factors, adjustment model 1 was adjusted for gender, age, and race, and adjustment model 2 added family income, education level, smoking status, alcohol status, PIR, physical activity level, hypertension, and diabetes as covariates to model 1. We conducted a linear trend test by using the median of FM from different body parts as a continuous variable in the model. To explore the nonlinear relationship between FM and LBP, we employed a generalized additive model for smooth curve fitting.^[27]^ Additionally, we conducted subgroup analyses to test the robustness and potential variations across different subgroups, as well as to assess their interactions. R 3.6.1 software (R Foundation for Statistical Computing, Vienna, Austria; https://www.rproject.org/) and EmpowerStats (https://www.empowerstats.com) were used for all analyses. *P* < .05 indicates a statistically significant difference.

In the MR analysis, the prepared IVs and outcome data were statistically analyzed using R software (version 4.1.3). The OR values and 95% CI were calculated through regression models including inverse-variance weighted (IVW), MR-Egger regression, weighted median, weighted median estimatorS, and simple mode to assess the causal relationship between trunk and lower limb FM and LBP.^[28,29]^ The IVW method, based on summarized genotype data, combines Wald estimates of causal effects from all IVs that meet the assumptions of the 3 hypotheses using a meta-analysis approach to obtain an overall unbiased estimate. The resulting causal relationship is relatively stable, making IVW the primary statistical method in this study. The MR-Egger regression uses the reciprocal of the variance of the outcome variable for weighted calculations. It includes an intercept term and does not exclude pleiotropy, allowing it to provide estimated results through the intercept after correcting for pleiotropy without being influenced by IV pleiotropy. The IVW method, although relatively reliable, excludes ineffective IVs and pleiotropic effects. In contrast, weighted median estimator can improve the estimation results of IVW even when nearly half of the IVs are ineffective, and it provides effect estimates that are consistent in direction. The weighted median method identifies multiple variables, weighting each SNP pair’s causal effect estimate by the number of SNPs in each cluster, to derive the estimate with the maximum weight. Since each SNP carries equal weight in the simple mode method, SNPs with similar values are grouped into clusters, and the cluster with the most SNPs is used to assess the causal relationship.

## 3. Results

### 3.1. Cross-sectional study by NHANES

In the NHANES 1999 to 2004 dataset, out of a total of 31,126 participants, after excluding those lacking data on FM, LBP, and related covariates, a total of 10,965 participants were included in this study (see Figure S2, Supplemental Digital Content, https://links.lww.com/MD/P672 for the participant selection process). Table 1 compares the baseline characteristics of the total sample, participants with LBP, and those non-LBP. The results show significant differences (*P* < .001) between participants with LBP and non-LBP in terms of gender, race, education level, PIR, activity level, smoking status, alcohol status, BMI, and prevalence of hypertension. In contrast, individuals with LBP were more likely to be female, of other Latino descent, have a high school education, low income, less frequent walking habits, be smokers, alcohol consumers, and have hypertension and diabetes. Additionally, without adjusting for covariates, there was a significant association (*P* < .001) between trunk FM, right leg FM, left leg FM, and LBP.

**Table 1:** The baseline characteristics of the study population.

Variable	Total (N = 10965)	Low back pain (N = 4511)	No low back pain (N = 6810)	*P*-value
Age (yr), mean (SD)	50.69 ± 18.55	51.02 ± 18.03	50.49 ± 18.86	.093*
Gender, N (%)				**<.001**
Male	5589 (50.97%)	1972 (47.46%)	3617 (53.11%)	
Female	5376 (49.03%)	2183 (52.54%)	3193 (46.89%)	
Race, N (%)				**<.001**
Mexican American	2404 (21.92%)	805 (19.37%)	1599 (23.48%)	
Other Hispanic	456 (4.16%)	191 (4.60%)	265 (3.89%)	
Non-Hispanic White	5688 (51.87%)	2284 (54.97%)	3404 (49.99%)	
Non-Hispanic Black	2065 (18.83%)	753 (18.12%)	1312 (19.27%)	
Other Race	352 (3.21%)	122 (2.94%)	230 (3.38%)	
Education, N (%)				**<.001**
Less than high school	3426 (31.24%)	1367 (32.90%)	2059 (30.23%)	
High school diploma (including GED)	2615 (23.85%)	1075 (25.87%)	1540 (22.61%)	
More than high school	4924 (44.91%)	1713 (41.23%)	3211 (47.15%)	
PIR, N (%)				**<.001**
<1.3	3040 (27.72%)	1272 (30.61%)	1768 (25.96%)	
≥1.3, <3.5	4290 (39.12%)	1641 (39.49%)	2649 (38.90%)	
≥3.5	3635 (33.15%)	1242 (29.89%)	2393 (35.14%)	
Activity, N (%)				**<.001**
Not walking regularly	2734 (24.93%)	1141 (27.46%)	1593 (23.39%)	
Do not have to carry	5809 (52.98%)	2098 (50.49%)	3711 (54.49%)	
Climb stairs or hills often	1673 (15.26%)	610 (14.68%)	1063 (15.61%)	
Beavy work or heavy loads	749 (6.83%)	306 (7.36%)	443 (6.51%)	
Smoke, N (%)				**<.001**
Now	2490 (22.71%)	1075 (25.87%)	1415 (20.78%)	
Former	2985 (27.22%)	1170 (28.16%)	1815 (26.65%)	
Never	5490 (50.07%)	1910 (45.97%)	3580 (52.57%)	
Alcohol, N (%)				**<.001**
Heavy	2089 (19.05%)	821 (19.76%)	1268 (18.62%)	
Moderate	1474 (13.44%)	574 (13.81%)	900 (13.22%)	
Mild	3558 (32.45%)	1250 (30.08%)	2308 (33.89%)	
Former	2266 (20.67%)	924 (22.24%)	1342 (19.71%)	
Never	1578 (14.39%)	586 (14.10%)	992 (14.57%)	
Hypertension, N (%)				**<.001**
Yes	4689 (42.76%)	1930 (46.45%)	2759 (40.51%)	
No	6276 (57.24%)	2225 (53.55%)	4051 (59.49%)	
Diabetes, N (%)				**.002**
DM	1487 (13.56%)	626 (15.07%)	861 (12.64%)	
IFG	411 (3.75%)	153 (3.68%)	258 (3.79%)	
NO	9067 (82.69%)	3376 (81.25%)	5691 (83.57%)	
BMI (kg/m^2^), mean (SD)	28.35 ± 6.23	29.08 ± 6.72	27.90 ± 5.86	**<.001** *
Left leg fat (kg), mean (SD)	4.72 ± 2.12	4.95 ± 2.25	4.57 ± 2.02	**<.001** *
Right leg fat (kg), mean (SD)	4.86 ± 2.19	5.10 ± 2.32	4.71 ± 2.09	**<.001** *
Trunk fat (Kg), mean (SD)	14.08 ± 6.42	14.89 ± 6.86	13.58 ± 6.09	**<.001** *

Bold values indicate statistically significant difference at *P* < .05

BMI = body mass index, DM = diabetes mellitus, IFG = impaired fasting glucose, PIR = poverty income ratio, SD = standard deviation.

* *P*-value: for continuous variables, the Kruskal–Wallis rank sum test was used, while for count variables with theoretical numbers <10, Fisher exact probability test was used to obtain the results.

**Figure 1.**
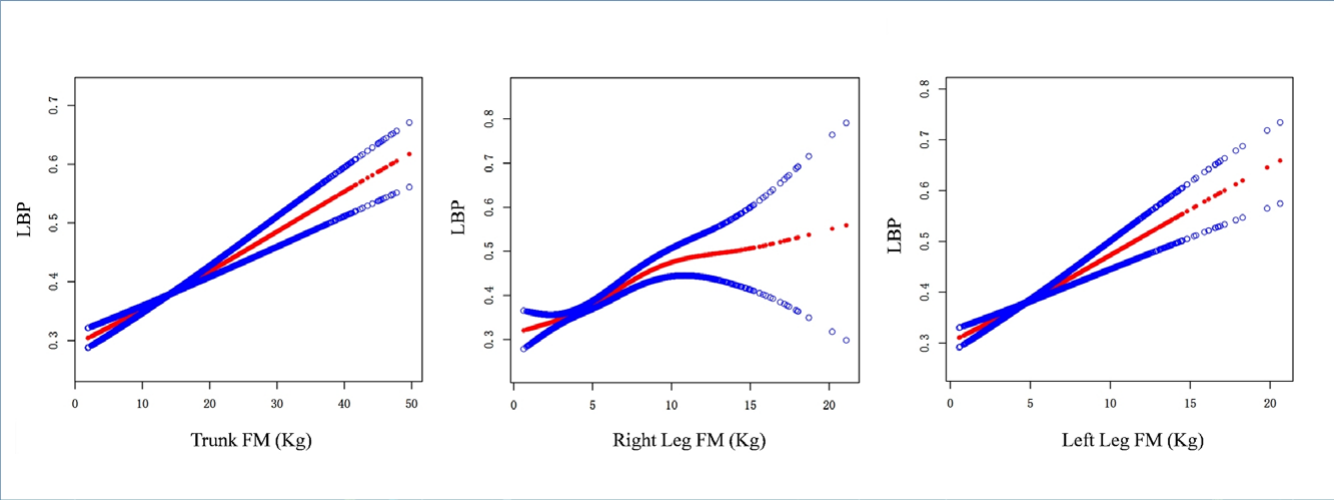
Smooth curve fitting of FM at different sites to LBP. The red line represents the smooth curve fitting between FM at different sites and LBP. The blue line represents the 95% confidence interval for the fit. The age, gender, race, education, PIR, activity, smoke, alcohol, hypertension and diabetes were adjusted. FM = fat mass, LBP = low back pain, PIR = poverty income ratio.

Table 2 presents the logistic regression models for different regions of FM and LBP. In the 3 adjusted models for trunk FM and LBP, there was a significant positive correlation between trunk FM and LBP. The results of the unadjusted model showed that for every one-unit increase in trunk FM (kg), the risk of LBP increased by 3.2% (OR = 1.032, 95% CI [1.026, 1.038], *P* < .00001). After adjusting for age, sex, and race only, each one-unit increase in trunk FM was associated with a 3.1% increase in the risk of LBP (OR = 1.031, 95% CI [1.024, 1.037], *P* < .00001). In the fully adjusted model, where all covariates were included, each one-unit increase in trunk FM was associated with a 2.8% increase in the risk of LBP (OR = 1.028, 95% CI [1.021, 1.034], *P* < .00001). To mitigate the impact of outliers on the results, trunk FM was categorized based on quartiles, and a trend test was conducted. The results showed a significant increase in the risk of LBP with increasing trunk FM in all 3 adjusted models (*P* for trend < .0001). Similarly, there was a positive correlation between right leg FM and left leg FM with LBP (fully adjusted model: OR = 1.075, 95% CI [1.053, 1.097], *P* < .00001; OR = 1.076, 95% CI [1.053, 1.099], *P* < .00001). Right leg FM and left leg FM were categorized based on quartiles, and a trend test was conducted. The results showed a significant increase in the risk of LBP for individuals in the highest quartile compared to the lowest quartile (fully adjusted model: OR = 1.517, 95% CI [1.337, 1.722], *P* < .00001; OR = 1.506, 95% CI [1.327, 1.710], *P* < .00001), with a significant trend (*P* for trend < .0001). To further explore the nonlinear dose–response relationship between FM at different body sites and LBP, we conducted smooth curve fitting. The results showed a nonlinear positive correlation between trunk FM, right leg FM, left leg FM, and LBP (see Fig. 1). We further performed subgroup analyses to investigate the association between trunk FM and LBP. The results revealed that the association between trunk FM and LBP was modified by race (*P* for interaction = .0403), income (*P* for interaction = .0244), activity level (*P* for interaction = .0034), alcohol consumption (*P* for interaction < .0001), and hypertension status (*P* for interaction = .0467) (see Table 3).

**Table 2:** The logistic regression analysis of the association between FM and LBP.

	Crude model	Adjusted model 1	Adjusted model 2
	OR (95% CI) *P*	OR (95% CI) *P*	OR (95% CI) *P*
Trunk FM (kg)	1.032 (1.026, 1.038) < **.00001**	1.031 (1.024, 1.037) < **.00001**	1.028 (1.021, 1.034)** < .00001**
Trunk FM (kg) quartile			
Q1	Reference	Reference	Reference
Q2	1.172 (1.048, 1.310) **.00538**	1.186 (1.059, 1.328) **.00325**	1.220 (1.088, 1.369) **.00069**
Q3	1.224 (1.095, 1.368) **.00036**	1.232 (1.099, 1.382) **.00036**	1.232 (1.096, 1.386) **.00047**
Q4	1.697 (1.521, 1.894) < **.00001**	1.670 (1.493, 1.868) < **.00001**	1.608 (1.430, 1.809) < **.00001**
*P* for trend	**<.0001**	**<.0001**	**<.0001**
Right leg FM (kg)	1.084 (1.065, 1.103) < **.00001**	1.076 (1.055, 1.097) < **.00001**	1.075 (1.053, 1.097) < **.00001**
Right Leg FM (kg) quartile			
Q1	Reference	Reference	Reference
Q2	1.164 (1.041, 1.300) **.00742**	1.121 (1.002, 1.255) **.04671**	1.164 (1.038, 1.305) **.00940**
Q3	1.149 (1.028, 1.284) **.01454**	1.072 (0.953, 1.207) .24485	1.115 (0.989, 1.257) .07617
Q4	1.618 (1.450, 1.805) < **.00001**	1.507 (1.333, 1.704) < **.00001**	1.517 (1.337, 1.722) < **.00001**
*P* for trend	**<.0001**	**<.0001**	**<.0001**
Left leg FM (kg)	1.086 (1.066, 1.106) < **.00001**	1.077 (1.055, 1.099) < **.00001**	1.076 (1.053, 1.099) < **.00001**
Left leg FM (kg) quartile			
Q1	Reference	Reference	Reference
Q2	1.193 (1.067, 1.333) **.00186**	1.149 (1.026, 1.286) **.01631**	1.197 (1.067, 1.343) **.00212**
Q3	1.202 (1.076, 1.343) **.00115**	1.123 (0.998, 1.263) .05430	1.169 (1.036, 1.318) **.01119**
Q4	1.604 (1.438, 1.790) < **.00001**	1.497 (1.324, 1.693) < **.00001**	1.506 (1.327, 1.710) < **.00001**
*P* for trend	**<.0001**	**<.0001**	**<.0001**

Bold values indicate statistically significant difference at *P* < .05.

Crude model: no covariates were adjusted.

Adjusted model 1: age, gender, and race were adjusted.

Adjusted model 2: age, gender, race, education, PIR, activity, smoke, alcohol, hypertension and diabetes were adjusted.

FM = fat mass, LBP = low back pain, OR = odds ratio, PIR = poverty income ratio.

**Table 3:** Relationship between Trunk FM and LBP in subgroups of potential effect modifiers.

Subgroup	OR	95% CI	*P*-value	*P* for interaction*
Gender				.0981
Male	1.022	(1.012, 1.032)	**<.0001**	
Female	1.033	(1.024, 1.042)	**<.0001**	
Age (yr)				.1587
<50	1.024	(1.016, 1.032)	**<.0001**	
≥50	1.033	(1.023, 1.043)	**<.0001**	
Race				**.0403**
Mexican American	1.042	(1.026, 1.058)	**<.0001**	
Other Hispanic	1.049	(1.012, 1.087)	**.0083**	
Non-Hispanic White	1.022	(1.013, 1.031)	**<.0001**	
Non-Hispanic Black	1.025	(1.013, 1.038)	**<.0001**	
Other Race	1.063	(1.026, 1.101)	**.0008**	
Education				.0844
Less Than High School	1.039	(1.027, 1.051)	**<.0001**	
High School Diploma (including GED)	1.021	(1.009, 1.034)	**.0008**	
More Than High School	1.025	(1.016, 1.034)	**<.0001**	
PIR				**.0244**
<1.3	1.04	(1.028, 1.052)	**<.0001**	
≥1.3, <3.5	1.027	(1.017, 1.038)	**<.0001**	
≥3.5	1.017	(1.006, 1.029)	**.0028**	
Activity				**.0034**
do not walk about very much	1.041	(1.030, 1.053)	**<.0001**	
do not have to carry	1.025	(1.016, 1.034)	**<.0001**	
climb stairs or hills often	1.022	(1.005, 1.039)	**.0122**	
heavy work or heavy loads	0.991	(0.965, 1.018)	.5097	
Smoke				.2655
Now	1.029	(1.016, 1.041)	**<.0001**	
Former	1.036	(1.024, 1.049)	**<.0001**	
Never	1.023	(1.014, 1.033)	**<.0001**	
Alcohol				**<.0001**
Heavy	1.023	(1.008, 1.037)	**.0022**	
Moderate	1.005	(0.989, 1.022)	.5227	
Mild	1.016	(1.004, 1.028)	**.0076**	
Former	1.048	(1.034, 1.062)	**<.0001**	
Never	1.048	(1.031, 1.065)	**<.0001**	
Hypertension				**.0467**
Yes	1.035	(1.025, 1.045)	**<.0001**	
No	1.022	(1.013, 1.031)	**<.0001**	
Diabetes				.0633
DM	1.046	(1.029, 1.062)	**<.0001**	
IFG	1.026	(0.995, 1.059)	.0958	
NO	1.024	(1.017, 1.032)	**<.0001**	

Adjusted for: gender, age, race, education, PIR, smoke, alcohol, activity, hypertension, diabetes. Bold values indicate statistically significant difference at *P* < .05.

BMI = body mass index, DM = diabetes mellitus, FM = fat mass, IFG = impaired fasting glucose, LBP = low back pain, OR = odds ratio, PIR = poverty income ratio.

* The *P* for interaction was additionally computed through the log-likelihood ratio test, which compared models that included and excluded the interaction of confounders.

### 3.2. MR analysis

**Figure 2.**
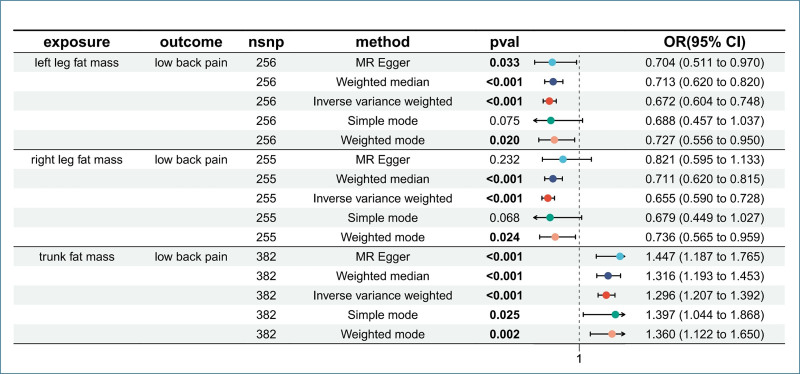
Forest plot for the causal effect of FM on the risk of LBP. Forest plot for the causal effect of FM on the risk of LBP derived from inverse-variance weighted (IVW), MR-Egger, weighted median (WME), simple mode (SM), weighted mode (WM). CI = confidence interval, FM = fat mass, LBP = low back pain, OR = odds ratio.

**Figure 3.**
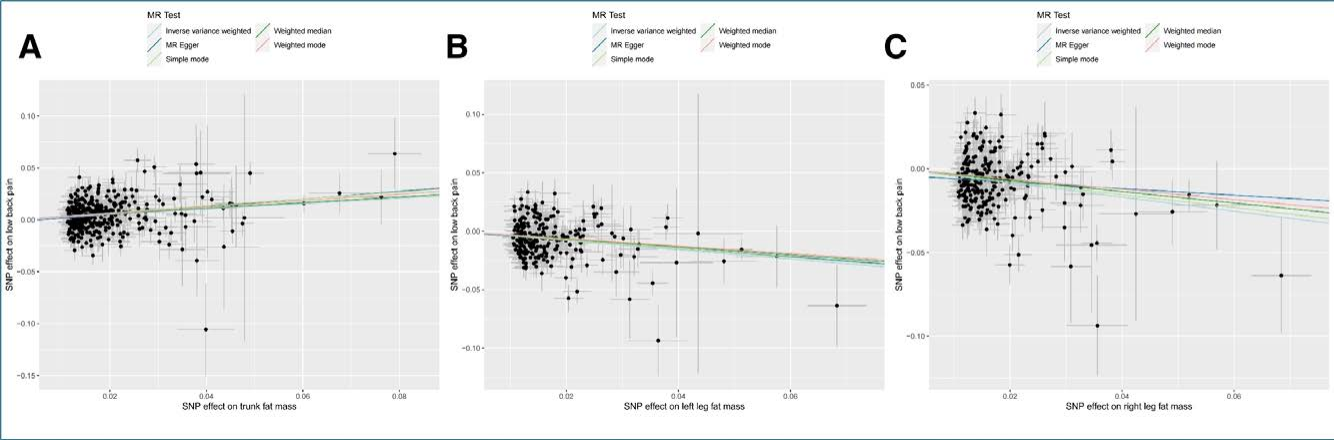
Scatter plots of Mendelian randomization tests. Scatter plots of Mendelian randomization tests assessing the effect of (A) trunk fat mass on LBP, (B) left leg fat mass on LBP, and (C) right leg fat mass on LBP. LBP low back pain.

In the MR analysis, after using the clumped function to remove linkage disequilibrium, we calculated that the F-values for each individual SNP in this study were all > 10 (trunk: 27.36–795.01, left leg: 18.18–419.18, right leg: 18.75–430.60), indicating the absence of weak instrument bias. Following the removal of palindromic single nucleotide polymorphisms, MR-PRESSO analysis detected no outliers, and the final IVs for each regional FM were summarized in the Supplementary Excel Table, Supplemental Digital Content, https://links.lww.com/MD/P674. The initial analysis using IVW indicated that an increase in trunk FM has a harmful effect on LBP (OR = 1.296, 95% CI [1.207, 1.392], *P* = 9.86 × 10^-13^), while an increase in FM in the left leg (OR = 0.672, 95% CI [0.604, 0.748], *P* = 2.61 × 10^-13^) and right leg (OR = 0.655, 95% CI [0.590, 0.728], *P* = 3.32 × 10^-15^) has a protective effect on LBP (Fig. 2). These results were consistent across all 5 MR methods in terms of the direction of the causal effect (Table S1, Supplemental Digital Content, https://links.lww.com/MD/P673), and scatter plots showed no significant deviation from the regression line (Fig. 3), indicating the reliability of the aforementioned causal relationships.

Sensitivity analysis using MR-Egger regression showed *P*-values > .05, indicating no evidence of potential horizontal pleiotropy. MR-PRESSO did not detect any outliers, indicating that the 3 IVs do not influence the outcome through pathways other than exposure, thus satisfying the exclusivity assumption. In the Cochran Q heterogeneity test for instrument variable compatibility (see Table S2, Supplemental Digital Content, https://links.lww.com/MD/P673), potential heterogeneity was observed for both trunk and lower limb FM. This may be attributed to variations in sample sizes, different covariate adjustments, diverse analytical methods, and varying databases. However, the IVW method’s data has clearly indicated a causal relationship, meeting the assumption of instrument independence. Therefore, the presence of heterogeneity is acceptable and does not affect the interpretation of positive results. Additionally, we found that in the leave-one-out analysis, when excluding individual SNPs, the risk estimates for SNPs remained robust (Figures S3–S5, Supplemental Digital Content, https://links.lww.com/MD/P672).

## 4. Discussion

In this study, we combined data from the NHANES database with MR methods to explore the potential relationship between FM in 3 body regions (trunk FM, right leg FM, left leg FM) and LBP. To our knowledge, this is the first study to combine a cross-sectional study with MR analysis to investigate the association between FM distribution and LBP. Through our research, we have uncovered some interesting findings.

Firstly, in the NHANES study, we found a significant positive correlation between trunk FM and LBP in 3 different adjustment models, whether trunk FM was considered as a continuous variable or a categorical variable. As trunk FM increased, the prevalence of LBP also increased, a conclusion further validated by MR analysis, indicating trunk FM as a potential risk factor for LBP. Additionally, after conducting subgroup analysis on the association between trunk FM and LBP, we observed significant differences in this association across different ethnicities, income levels, activity levels, alcohol consumption levels, and hypertension status.

In the results showing an increased risk of LBP with higher trunk FM, we believe that besides the excessive mechanical stress it brings, the substances released by excessive fat are also among the important factors affecting intervertebral discs.^[30]^ The mechanism by which obesity causes bone and joint-related diseases may involve the toxic effects of lipids, specifically the accumulation of free fatty acids in non-adipose tissues.^[31,32]^ In pathological conditions, adipose tissue releases adipocyte-derived factors that not only participate in energy metabolism but also induce the liver to produce pro-inflammatory factors. These inflammation-related factors may lead to changes in the inflammatory environment of the spine, causing local pain.^[33,34]^ For example, Jamaluddin et al found that resistin induces an inflammatory environment and regulates angiogenesis in joint parts through the NF-κB pathway. Its levels are positively correlated with Pfirrmann grades and can also modulate macrophages to some extent, thereby influencing the severity of inflammation.^[35]^ In addition to the release of adipokines, the influence of trunk FM on LBP may also be related to fat infiltration. The multifidus muscle in the lumbar region plays a primary role in maintaining the stability of the lumbar spine by resisting spinal rotation and movement. When the multifidus muscle experiences a reduction in strength due to infiltration by adipose tissue, its stabilizing function is compromised. This leads to a diminished ability to control external loads such as shear and sliding forces, resulting in an inability to maintain the structural stability of the intervertebral discs and thus contributing to LBP. In addition, an article published in *Pain* suggests that while there is an association between inflammation and LBP outcomes, the biological markers behind inflammation may vary due to racialization.^[36]^ Furthermore, epidemiological studies have indicated that LBP is a major cause of disability in low- and middle-income countries, possibly due to the fact that low-income populations often engage in strenuous labor.^[37]^ Reports have also indicated that individuals with back pain are more likely to have dependencies on alcohol, cannabis, and other substances. A nationwide study in the United States found that excessive alcohol consumption is associated with the occurrence of gout, rheumatoid arthritis, fibromyalgia, osteoarthritis, or LBP.^[38,39]^ The conclusions from these studies are similar to the results of our subgroup analysis.

The previous studies that focused on categorizing FM into trunk and lower extremities primarily targeted internal medicine diseases. For instance, Snijder et al suggested that in women, accumulation of fat in the legs seems to prevent disruptions in glucose metabolism, which is closely related to the incidence of cardiovascular and metabolic diseases.^[40,41]^ Other studies have indicated that fat distribution, rather than total FM, is more predictive of individual insulin resistance and related complications.^[42]^ The specific mechanisms underlying fat distribution and quantity in the field of musculoskeletal diseases remain unclear. Investigating the risk of disease development based on different regional fat distributions and establishing more reliable assessment criteria to guide clinical practice hold significant clinical significance for future research.^[43]^

After studying the correlation between lower extremity FM and LBP, we found discrepancies between the results of cross-sectional studies and MR analysis. In cross-sectional studies, both right leg FM and left leg FM showed a positive correlation with LBP, indicating that an increase in lower extremity FM could increase the likelihood of developing LBP. However, MR analysis revealed that an increase in lower extremity FM has a protective effect against LBP. We believe that this difference may be related to the classification of obese populations. Based on the site of fat accumulation, obesity can be classified into central obesity (abdominal obesity) and peripheral obesity (subcutaneous fat obesity). Central obesity is characterized by the predominant accumulation of fat in the abdomen, increased visceral fat, thickening of the waist, and a “pear-shaped” obesity pattern. Patients with this type of obesity are more prone to metabolic diseases such as diabetes. Peripheral obesity, on the other hand, is characterized by fat accumulation in the thighs, buttocks, and other areas, presenting an “apple-shaped” obesity pattern. A meta-analysis published in BMJ indicates that most indices of central obesity, including waist circumference, waist-to-hip ratio, waist-to-height ratio, waist-to-thigh ratio, and BMI, are positively correlated with increased risk of all-cause mortality.^[44]^ Furthermore, the study points out that an increase in thigh circumference and hip circumference can reduce the risk of all-cause mortality, which aligns with the conclusions drawn from our MR analysis. Additionally, the article supports that this correlation is more significant in females. However, in our cross-sectional study, we were unable to differentiate between central obesity and peripheral obesity patients, leading to difficulties in avoiding the confounding effects of central obesity on the correlation between lower limb FM and LBP. Additionally, we believe that increased FM in the lower limbs may influence the loading on intervertebral discs through changes in human behavior, which could have a protective effect on the structure of intervertebral discs.^[45]^ A study on elderly individuals suggests that lower limb muscle mass may be a crucial determinant of gait function.^[46]^ Therefore, changes in lower limb FM might affect daily activities like gait and posture, potentially reducing the risk of LBP. However, this area requires further exploration and additional studies for thorough analysis. Previous research on lower limb fat and LBP is limited, but incorporating it as a protective factor in evaluating the impact of obesity on LBP could introduce new hypotheses and research directions, ultimately offering better treatment and prevention strategies.

Different distributions of FM increasing the risk of LBP or potentially acting as protective factors cannot be conclusively determined solely based on observational studies. In addition to conducting a cross-sectional study using the NHANES database, this study utilized MR as a novel epidemiological research method, which offers several advantages: (1) genetic variations selected as IVs are formed independently of social, lifestyle, and other confounding factors, leading to more reliable results. (2) Genetic variations are based on Mendel laws, preceding the exposure factors and outcome variables, thus avoiding the possibility of reverse causation in MR analysis. (3) Data for MR analysis are sourced from publicly available literature and databases, maintaining a degree of randomness akin to randomized controlled trials, and ensuring trustworthy results. But this study still has limitations. Firstly, the use of NHANES database along with data from the UK Biobank and Finnish databases is primarily based on populations from South America and Europe, which may limit the generalizability of the conclusions to other populations. The lack of data from Asian populations and other regions is a significant factor limiting the external validity of the study findings. Secondly, despite accounting for covariates in the cross-sectional study, it is still unable to fully eliminate the potential influence of all confounding factors as mentioned earlier. And furthermore, the proportion of abdominal and hip fat accumulation differs between males and females, leading to varying shear forces on the lumbar spine.^[47]^ This study did not further explore gender differences, and only categorized FM into trunk and lower limbs without specific differentiation of the abdomen and lumbar back. This limitation is due to the inability to obtain more precise patient data from various databases. Finally, the validation section of this study demonstrated heterogeneity in the results. In the MR analysis, despite filtering based on a *P*-value threshold of 5 × 10^-8^ and the average F-value, the results still retained a large number of SNPs. This suggests that the excessive number of SNPs may be the primary source of heterogeneity in this study. But, after the rigorous screening process, the SNPs finally included in the analysis were strong IVs and excluded horizontal pleiotropy to avoid bias. Considering the relevant literature previously published,^[48]^ it can be argued that the heterogeneity in this study does not affect the interpretation of the research results.

## 5. Conclusions

In summary, this study combined NHANES database with MR analysis to reveal a positive causal association between trunk FM and LBP, while the causal relationship between lower limb FM and LBP remains somewhat controversial. However, investigating lower limb FM as a potential protective factor warrants further exploration. This study conducted dual analyses using large-sample clinical data and genetic data focusing on FM and its primary distribution, providing a new perspective on the impact of obesity on LBP. It suggests that in future clinical settings, more attention should be given to FM and its distribution when treating and preventing LBP.

## Author contributions

**Data curation:** Jingyan Yang, Yupei Cheng, She Ma, Jingjie Huang.

**Funding acquisition:** Dong Yu.

**Methodology:** Jingyan Yang, Yupei Cheng.

**Project administration:** Bangqi Wu.

**Supervision:** Bangqi Wu, Dong Yu.

**Software:** Renjun Huang, Chaoran Wang.

**Validation:** Yuyang Zhao, Chaoyi Wang, Qian Yu.

**Visualization:** Yupei Cheng, Yuyan Chen, Chaoran Wang.

**Writing – original draft:** Jingyan Yang, Yupei Cheng.

**Writing – review & editing:** Bangqi Wu, Dong Yu.

## Supplementary Material


