# Supplementary material for: The potential causal relationship between fat mass in different body areas and low back pain: Findings from NHANES and Mendelian randomization studies
Source: Medicine (Baltimore). 2025 Aug 15;104(33):e43891. doi: 10.1097/MD.0000000000043891 (PMC12366917; doi:10.1097/MD.0000000000043891)
Supplement: Supplementary file 1 [file medi-104-e43891-s001.docx]

***Supplementary Figure S1***


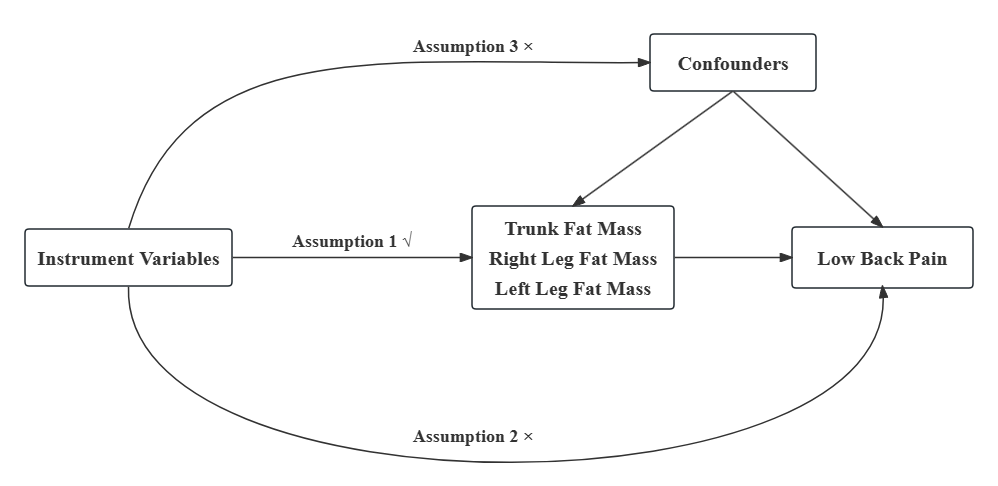


**Figure S1** The research is based on three hypotheses: (1) The instrumental variable is strongly correlated with Trunk Fat Mass, Right Leg Fat Mass and Left Leg Fat Mass; (2) The instrumental variable is not correlated with the confounding factors; (3) The instrumental variable is not directly related to Low Back Pain, and its effect on Low Back Pain can only be through Fat Mass to reflect.

***Supplementary Figure S2***


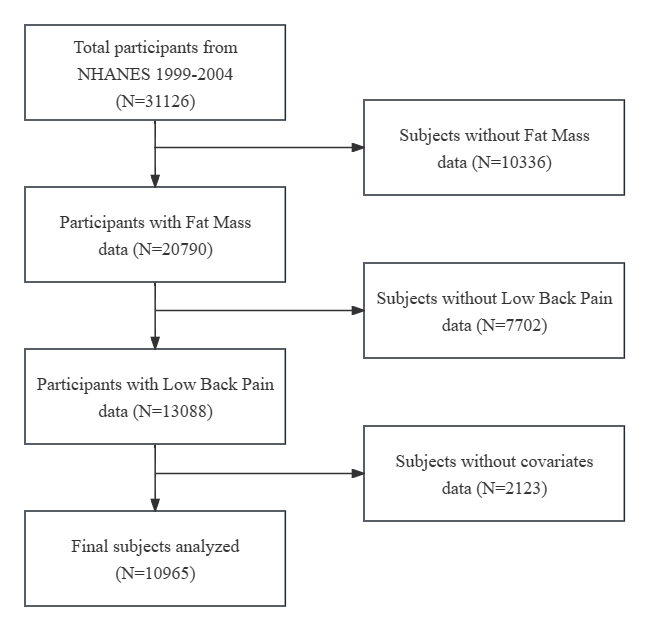


**Figure S2 Flowchart of participants selection.**

***Supplementary Figure S3***


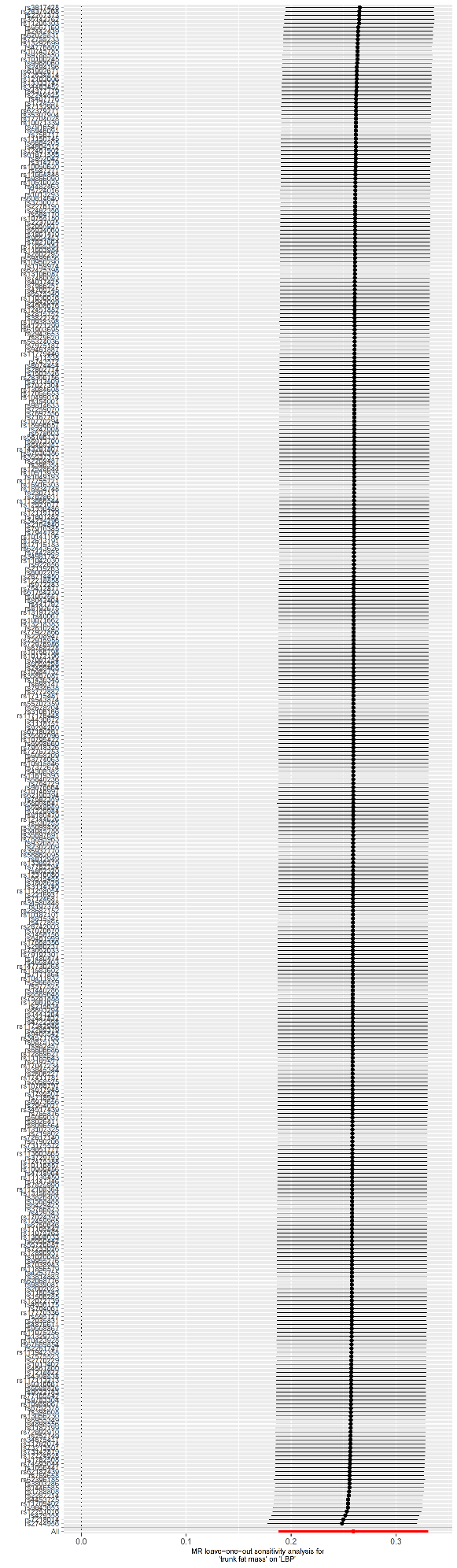


**Figure S3 Results of leave-one-out sensitivity analysis for trunk fat mass on LBP.**

***Supplementary Figure S4***


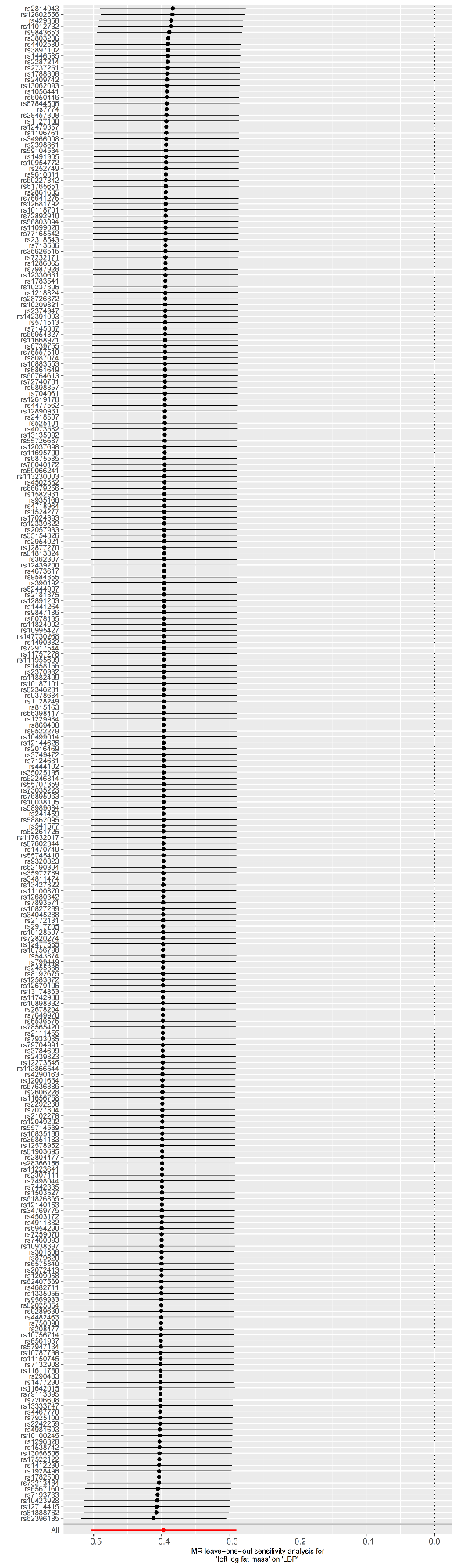


**Figure S4 Results of leave-one-out sensitivity analysis for left leg fat mass on LBP.**

***Supplementary Figure S5***


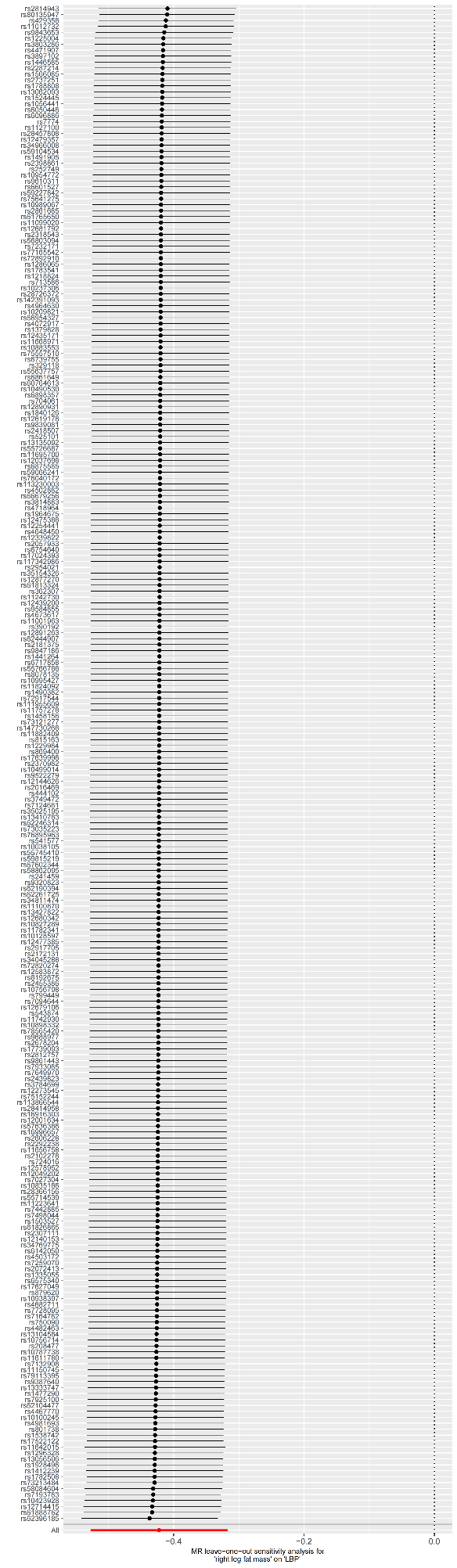


**Figure S5 Results of leave-one-out sensitivity analysis for right leg fat mass on LBP.**
