# Supplementary material for: The potential causal relationship between fat mass in different body areas and low back pain: Findings from NHANES and Mendelian randomization studies
Source: Medicine (Baltimore). 2025 Aug 15;104(33):e43891. doi: 10.1097/MD.0000000000043891 (PMC12366917; doi:10.1097/MD.0000000000043891)
Supplement: Supplementary file 3 [file medi-104-e43891-s003.docx]

***Supplementary Table S1***

| Outcome | Exposure | Method | nsnp | OR(95%CI) | *P* | Lower CI | Upper CI | OR | Lower CI | Upper CI |
| --- | --- | --- | --- | --- | --- | --- | --- | --- | --- | --- |
| Low back pain | Trunk fat mass | MR Egger | 382 | 1.447（1.187-1.765） | 0.29×10^-3^ | 0.171 | 0.568 | 1.447 | 1.187 | 1.765 |
|  |  | Weighted median | 382 | 1.316（1.193-1.453） | 4.92×10^-8^ | 0.176 | 0.374 | 1.316 | 1.193 | 1.453 |
|  |  | Inverse variance weighted | 382 | 1.296（1.207-1.392） | 9.86×10^-13^ | 0.188 | 0.331 | 1.296 | 1.207 | 1.392 |
|  |  | Simple mode | 382 | 1.397（1.044-1.868） | 0.025 | 0.044 | 0.625 | 1.397 | 1.045 | 1.868 |
|  |  | Weighted mode | 382 | 1.360（1.122-1.650） | 0.002 | 0.115 | 0.501 | 1.360 | 1.122 | 1.650 |
|  | Left leg fat mass | MR Egger | 256 | 0.704（0.511-0.970） | 0.033 | -0.672 | -0.029 | 0.704 | 0.511 | 0.97 |
|  |  | Weighted median | 256 | 0.713（0.620-0.820） | 2.15×10^-6^ | -0.477 | -0.198 | 0.713 | 0.620 | 0.82 |
|  |  | Inverse variance weighted | 256 | 0.672（0.604-0.747） | 2.61×10^-13^ | -0.504 | -0.291 | 0.672 | 0.604 | 0.748 |
|  |  | Simple mode | 256 | 0.688（0.457-1.037） | 0.075 | -0.783 | 0.036 | 0.688 | 0.457 | 1.037 |
|  |  | Weighted mode | 256 | 0.726（0.556-0.949） | 0.02 | -0.587 | -0.052 | 0.727 | 0.556 | 0.949 |
|  | Right leg fat mass | MR Egger | 255 | 0.821（0.595-1.132） | 0.232 | -0.518 | 0.125 | 0.821 | 0.595 | 1.133 |
|  |  | Weighted median | 255 | 0.711（0.620-0.815） | 9.81×10^-7^ | -0.477 | -0.204 | 0.711 | 0.620 | 0.815 |
|  |  | Inverse variance weighted | 255 | 0.655（0.590-0.728） | 3.32×10^-15^ | -0.528 | -0.317 | 0.655 | 0.590 | 0.728 |
|  |  | Simple mode | 255 | 0.679（0.449-1.026） | 0.068 | -0.799 | 0.026 | 0.679 | 0.449 | 1.027 |
|  |  | Weighted mode | 255 | 0.736（0.565-0.958） | 0.024 | -0.57 | -0.042 | 0.736 | 0.565 | 0.959 |

**Table S1:** Associations between genetically predicted increase in fat mass in different distributions and low back pain in Mendelian Randomization analyses.

***Supplementary Table S2***

| Exposure | Outcome | Q | Q-df | *P*-Het | Egger-intercept | Egger-SE | *P*-Egger |
| --- | --- | --- | --- | --- | --- | --- | --- |
| Trunk fat mass | LBP | 648.165 | 381 | 3.57x10^-16^ | -0.002 | 0.002 | 0.244 |
| Left leg fat mass | LBP | 517.103 | 255 | 5.78x10^-20^ | -0.001 | 0.003 | 0.765 |
| Right leg fat mass | LBP | 512.731 | 254 | 1.24x10-^19^ | -0.004 | 0.003 | 0.147 |

**Table S2:** Sensitivity analysis of fat mass in different distributions on low back pain.

(Note: ‘*P*-Het’represents the *P*-value belonging to the Q-statistic. ‘*P*-Egger’ represents the *P*-value belonging to the Egger-intercept.)
